# Effectiveness of Protected Areas in Maintaining Plant Production

**DOI:** 10.1371/journal.pone.0019116

**Published:** 2011-04-28

**Authors:** Zhiyao Tang, Jingyun Fang, Jinyu Sun, Kevin J. Gaston

**Affiliations:** 1 Department of Ecology, College of Urban and Environmental Sciences and MOE Laboratory for Earth Surface Processes, Peking University, Beijing, China; 2 Biodiversity and Macroecology Group, Department of Animal and Plant Sciences, University of Sheffield, Sheffield, United Kingdom; University of Glamorgan, United Kingdom

## Abstract

Given the central importance of protected area systems in local, regional and global conservation strategies, it is vital that there is a good understanding of their effectiveness in maintaining ecological functioning. Here, we provide, to our knowledge, the first such global analysis, focusing on plant production, a “supporting” ecosystem function necessary for multiple other ecosystem services. We use data on the normalized difference vegetation index (NDVI) as a measure of variation in plant production in the core, boundary and surroundings of more than 1000 large protected areas over a 25 year period. Forested protected areas were higher (or similar), and those non-forested were lower (or similar), in NDVI than their surrounding areas, and these differences have been sustained. The differences from surrounding areas have increased for evergreen broadleaf forests and barren grounds, decreased for grasslands, and remained similar for deciduous forests, woodlands, and shrublands, reflecting different pressures on those surroundings. These results are consistent with protected areas being effective both in the representation and maintenance of plant production. However, widespread overall increases in NDVI during the study period suggest that plant production within the core of non-forested protected areas has become higher than it was in the surroundings of those areas in 1982, highlighting that whilst the distinctiveness of protected areas from their surroundings has persisted the nature of that difference has changed.

## Introduction

**Figure 1:**
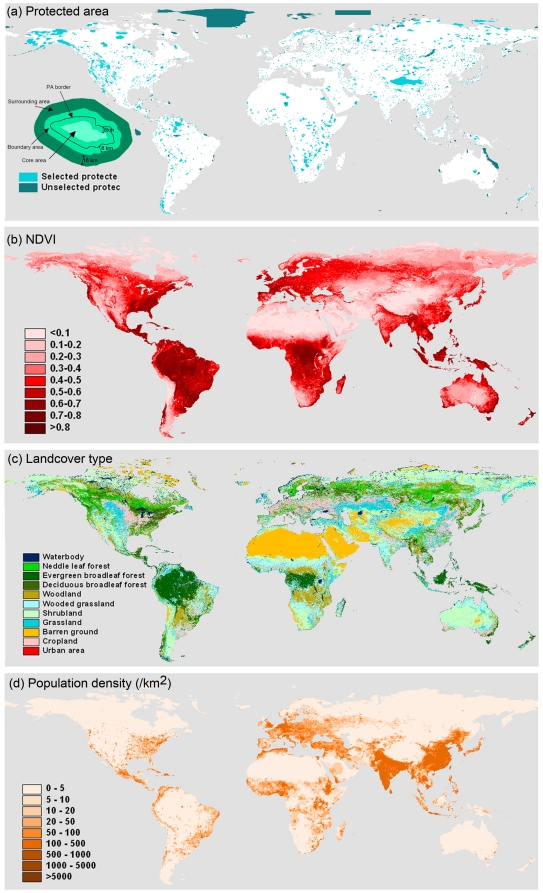
Global distribution. Distribution of (a) protected areas, (b) mean normalized difference vegetation index in the year 2006, (c) land-cover types, and (d) human population density in the year 2000. The insert in (a) illustrates the method used to buffer the protected areas.

Central to the vast majority of local, regional and global strategies for biological conservation, approximately one eighth of the Earth's terrestrial surface (∼12%) has now been formally designated as protected areas [1]–[2] (Fig. 1a). This is substantial, with by comparison agriculture (cropland and pasture), for example, extending over 40% of the ice-free surface [3]–[4]. The costs of this protected area system have thus been significant, in terms not only of identifying, establishing and managing the more than 100,000 sites of which it comprises, but also in the resultant lost or constrained opportunities for other land uses [5]–[9]. It is therefore vital to understand the contribution which this system actually makes to biological conservation [10]–[11].

Two components of the ecological effectiveness of protected areas are typically differentiated [10]–[12]. The first, representation or inventory, concerns the capture by protected areas of biodiversity features (e.g. biomes, ecosystems, habitats, species), especially those which are rare or threatened, and ecological (and perhaps evolutionary) processes (e.g. natural disturbance, biotic interactions, biogeochemical cycling, community succession). A wide variety of studies have attempted to assess how well regional and global protected area systems perform in this regard, predominantly in the context of ‘gap analyses’, where the focus is foremost on identifying those biodiversity features which are inadequately covered relative to specified targets [13]–[19]. These gaps tend to attract much attention, given their obvious implications for the expansion or realignment of protected area systems, with evidence of significant biases in the spatial and environmental distribution of these systems and of their failure to meet objective goals for the representation of features. Equally, however, such analyses also commonly document the occurrence of high proportions of regional and global sets of biodiversity features within protected areas, and in some regions the high proportions that are entirely or largely dependent on those areas [11], [18].

The second component of the ecological effectiveness of protected areas, persistence or condition, concerns how well these areas maintain the biodiversity features which occur and the ecological/evolutionary processes which take place within their bounds. In other words, to what extent these features and processes are actually protected or conserved. Empirical studies of this issue are far scarcer than are those for representation, and typically rather more limited in scope. In particular, they tend to be:

(1) based on space-for-time swaps, in which comparisons of the state of given biodiversity features or processes are made between the inside and outside of protected areas on the assumption that any differences are indicative of the consequences of protection [20]–[22]. Direct studies of temporal trends in biodiversity features and processes within protected areas are unusual [23]–[24];

(2) conducted at the scale of particular individual protected areas rather than that of protected area systems [25]–[27], limiting the generalizations that can be drawn given that those areas chosen for study are unlikely to comprise a strictly random sample; and

(3) focussed foremost on the success or otherwise of protected areas in maintaining the extent of particular land cover types, and especially forest cover [22], [27]–[31].

These biases continue severely to limit the extent to which general conclusions can be drawn about the persistence component of the ecological effectiveness of protected areas. Especially valuable would be studies that bear on the maintenance of the ecosystem functioning and services that inevitably underpin the persistence of biodiversity features [32]–[33]. Changes in land cover provide some indications of this, but substantial changes in ecosystem functioning may take place (e.g. through selective logging or intensive grazing) without necessarily markedly influencing such measures, especially when they are based on remote sensing imagery [34]–[40].

In this paper we use a rather different approach to the persistence component of the ecological effectiveness of protected areas to that which has typically been previously employed. Taking a global view, we examine variation in plant production, a major ecosystem function and ‘supporting’ ecosystem service (those necessary for the production of all other ecosystem services)[41], for 1015 large protected areas (each ≥500 km^2^; Fig. 1a) over a 25 year period (1982–2006). This is done within the core of each protected area (≥∼8 km inside the perimeter), within its boundary (∼8 km within to ∼8 km outside the perimeter), and in the surroundings (∼8 to 24 km outside the perimeter), enabling both spatial and genuinely temporal (rather than space-for-time swap) comparisons of plant production.

Plant production might differ between protected areas and their surroundings for a variety of reasons. These include (1) non-randomness in where protected areas were originally designated, resulting in initial production being different; (2) temporal changes in production within protected areas (e.g. from active management); and/or (3) temporal changes in production outside protected areas (e.g. through habitat loss and change) [11]. Although it can sometimes be helpful to attempt a fuller disaggregation of these factors, we regard non-randomness in the spatial location of protected areas, and any resultant differences in their initial plant production, as important determinants of their effectiveness (and of that of conservation planning), rather than confounding biases to be controlled for [22], [42]. We thus focus on the relative changes between protected areas and their surroundings. Indeed, because many large protected areas were designated before it was possible to estimate plant production from remote imagery, initial conditions are often impossible formally to determine in this regard (as they are for many other ecological variables).

## Materials and Methods

### Data

#### Protected areas

Analyses were based on the World Database on Protected Areas (WDPA) 2007, the most comprehensive global catalogue of protected areas, assembled by a broad alliance of organizations working in coordination with the IUCN World Commission on Protected Areas [43] (Fig. 1a). We used only those terrestrial protected areas designated as nature reserves, with an area of at least 500 km^2^. Marine, lake, and river protected areas were excluded, as were those without polygons and only recorded as points.

#### NDVI

Following previous studies in other contexts, we use the normalized difference vegetation index (NDVI; calculated from spectral reflectance measurements in the red and near-infrared regions), a variable linearly related to the fraction of photosynthetically active radiation absorbed by vegetation (fPAR), as a relative measure of plant production [44]–[49]. In linear combination with photosynthetically active radiation (PAR) and conversion efficiency, NDVI is commonly used to estimate gross primary production (GPP) at large scales [50]–[53]. In comparing each protected area and its surroundings, PAR is maintained as approximately constant, enabling NDVI to be used as a relative measure of GPP. NDVI data from the GIMMS-NDVI (Global Inventory Modeling and Mapping Studies) project for the period 1982–2006 were obtained from the Global Land Cover Facility [54]–[55]. This is calculated as (NIR -R)/(NIR+R), where NIR and R are the reflectance in the near infrared (0.725–1.l µm) and red (0.58–0.61 µm) wavebands measured by the AVHRR (Advanced Very High Resolution Radiometer) sensor of the NOAA (National Oceanic and Atmospheric Administration) satellites, and with the radiometric, atmospheric, cloud and stratospheric aerosol errors calibrated. It has an 8×8 km resolution (at the equator) and is composed of the maximum NDVI values for half-monthly periods. We used annual mean NDVI value (Fig. 1b).

#### Land cover

We used the UMD global land-cover classification data [56]–[57] to identify the biome type of each protected area. This was generated using imagery from AVHRR satellites acquired between 1981 and 1994, with a spatial resolution of 1×1 km. Fourteen land-cover classes are recognised, but we combined evergreen needleleaf and deciduous needleleaf forest, open shrub and closed shrub, and deciduous broadleaf forest and mixed forest, respectively (Fig. 1c).

#### Population density

We used the Gridded Population of the World Version 3 for the year 2000 to calculate the population density of each protected area and its surrounding areas [58]. These data have a spatial resolution of ∼4×4 km; to compare with NDVI, we resampled the resolution to 8×8 km (Fig. 1d).

### Data analysis

#### Buffering analysis

By applying buffer analysis in the geographic information system (GIS) software ArcView GIS 3.2 (ESRI, 1999), we buffered each of the protected areas into three zones, a core area ∼8 km (1 pixel of NDVI) inside the perimeter, a boundary area between ∼8 km within and ∼8 km outside the perimeter, and a surrounding area between ∼8 and ∼24 km outside the perimeter (Fig. 1a). The overlaps of surrounding buffers with core and boundary of neighbouring protected areas were excluded.

#### Zonal analysis

By applying zonal analysis in ArcView GIS 3.2, we calculated mean annual NDVI for the core and boundary of all protected areas and their surroundings for the period 1982–2006. We overlaid the protected area with population density and land-cover data to derive mean population density and composition of land-cover types within and around each protected area. We assigned protected areas based on dominant land-cover type within their bounds.

#### Statistical analysis

A paired sample t-test was first conducted to evaluate if NDVI is different between the core areas of the protected areas and their surroundings. A standard analysis of variance (ANOVA) was then performed to determine whether σNDVI (the differences in NDVI between the cores and their surroundings) were significantly different among land cover types. A least significance difference (LSD) test for all pairwise comparisons was conducted when a significant difference in the ANOVA was indicated (p≤0.05). A simple regression was then applied to explore the temporal trend of NDVI and σNDVI during 1982–2006 for the protected areas overall, for those that were forested/non-forested, as well as for protected areas of different land cover types.

## Results

### Averaged differences

**Figure 2:**
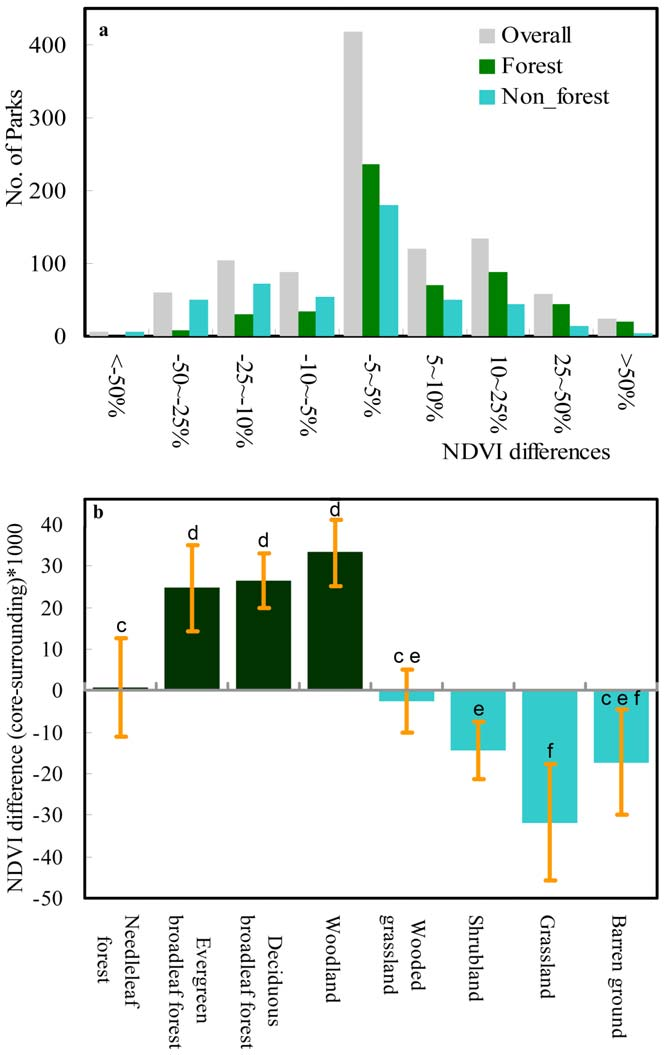
The time-averaged σNDVI (difference of NDVI between core of protected areas and their surroundings). (a) The number of protected areas with different levels of difference and (b) the difference for protected areas with different land cover types (bars indicate standard deviation). Green for forested, and cyan for non- forested protected areas. Different letters in (b) denote significant differences in σNDVI among land cover types.

**Figure 3:**
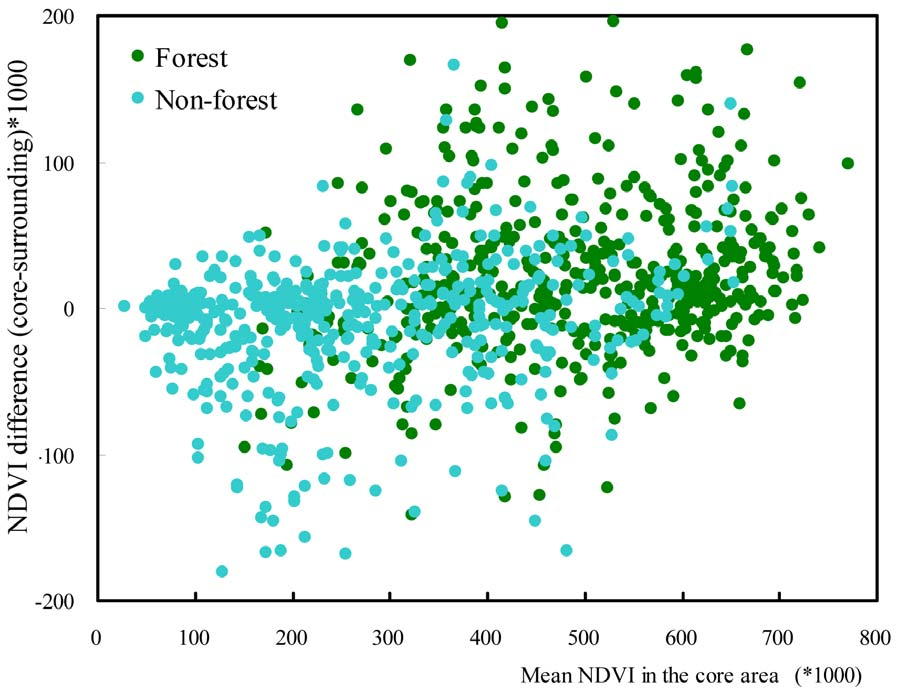
The relationship between δ-NDVI (difference of NDVI between core of protected areas and their surroundings) and average NDVI in the core areas (R^2^ = 0.11, p<0.001). Green for forested (R^2^ = 0.08, p<0.001), and cyan for non-forested (R^2^ = 0.07, p<0.001) protected areas.

The 25-year averaged NDVI for all 1015 protected areas was 1.6% higher (t = 3.11, n = 1015, p<0.001) in their core areas than in their surroundings. For 337 (33.2%) protected areas it was at least 5% higher, for 418 (41.2%) it was similar (−5% to +5%), and for 260 (25.6%) it was at least 5% lower (Fig. 2a). However, unsurprisingly but more importantly, the pattern differed between land cover types (F = 21.35, p<0.001; Fig. 2b). Of the 550 forest protected areas, for 230 (41.8%) NDVI was at least 5% higher in the core than in their surrounding areas, for 240 (43.6%) it was similar (−5∼5%), and for only 80 (14.6%) was it at least 5% lower. By contrast, for the 465 non-forested protected areas the numbers were 107 (23.0%), 178 (38.3%) and 180 (38.7%), respectively (Fig. 2a). In more detail, for broadleaf forest and woodland dominated protected areas NDVI was higher in the core of protected areas than in the surroundings (Fig. 2b), and for shrublands, grasslands and deserts (barren grounds) NDVI was lower in protected areas than in their surroundings (Fig. 2b). There were no significant differences in NDVI between protected areas and their surroundings for needleleaf forests and wooded grasslands (Fig. 2b). In general, the differences in NDVI between protected areas and their surroundings were positively correlated with the average NDVI in the core areas (Fig. 3).

### Temporal trends

**Figure 4:**
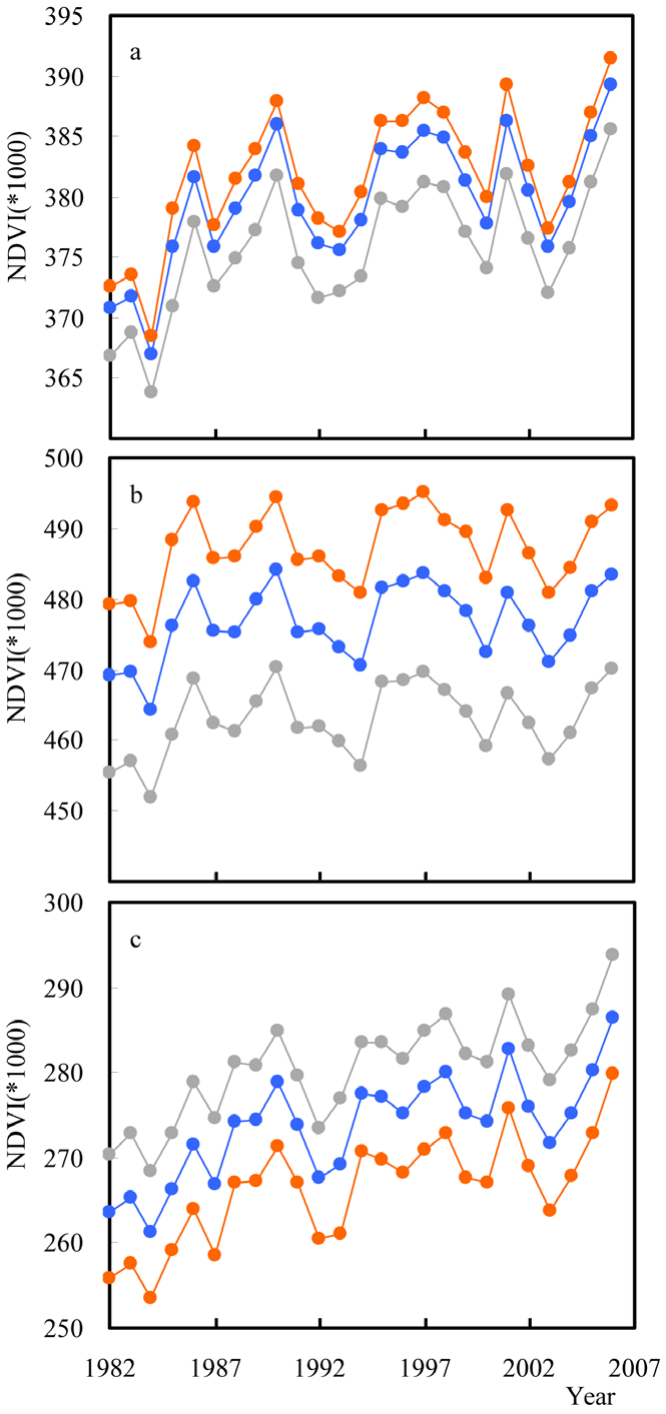
Temporal trends in the mean NDVI of the core of protected areas, their boundary and surroundings. (a) all protected areas for the core (orange; slope = 0.453×10^−3^, R^2^ = 0.36), boundary (blue; slope = 0.451×10^−3^, R^2^ = 0.38) and surroundings (grey; slope = 0.446×10^−3^, R^2^ = 0.40); (b) forested protected areas (core area: slope = 0.259×10^−3^, R^2^ = 0.12; boundary area: slope = 0.271×10^−3^, R^2^ = 0.14; and surrounding area: slope = 0.264×10^−3^, R^2^ = 0.14), and (c) non-forested protected areas (core area: slope = 0.652×10^−3^, R^2^ = 0.56; boundary area: slope = 0.633×10^−3^, R^2^ = 0.57; and surrounding area: slope = 0.637×10^−3^, R^2^ = 0.60).

From 1982 to 2006 annual NDVI increased significantly in the core and boundary of protected areas and in their surroundings, by an average of ∼2.3%. NDVI increased in the core areas of 385 (37.9%) protected areas, decreased in 85 (8.4%), and showed no significant trend in 545 (53.7%) (Fig. 4a). For the forest protected areas the respective numbers were 159 (28.9%), 68 (12.4%) and 323 (58.7%), and for the non-forested protected areas they were 226 (48.6%), 17 (3.6%) and 222 (47.7%) (Fig. 4b & c).

Importantly, these changes have been sufficient that in non-forested protected areas, although on average NDVI in surrounding areas has remained consistently higher than in the core of protected areas, in 2006 it was at a substantially higher level in the core than it was in the surroundings in 1982 (Fig. 4c).

**Figure 5:**
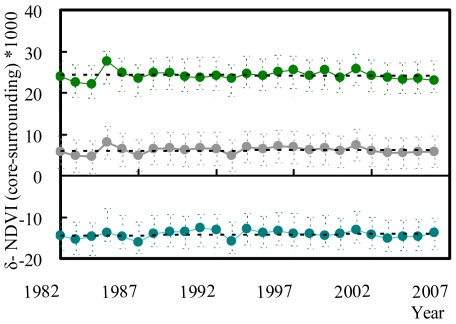
Temporal trends in mean δ-NDVI (difference of NDVI between the core of protected areas and their surroundings). Grey for all, green for forested, and cyan for non-forested protected areas.

Overall, there were no trends through time in the difference in NDVI between protected areas and their surroundings (Fig. 5). The difference increased for 147 (14.5%) protected areas, decreased for 138 (13.6%), and did not vary significantly for 730 (71.9%). For the forest protected areas the equivalent numbers were 79 (14.4%), 69 (12.5%) and 402 (73.1%), and for non-forested protected areas they were 68 (14.6%), 69 (14.8%), and 328 (70.6%). These patterns were broadly similar for the more detailed breakdown of land-cover types (Fig. S1). However, there were net temporal increases in the difference in NDVI between protected areas and their surroundings for evergreen broadleaf forests and barren grounds (Fig. S2).

## Discussion

The ecological effectiveness of protected areas has previously been rather poorly explored, particularly at the level of protected area systems (rather than given individual protected areas), using genuinely temporal data (rather than space-for-time swaps), and using measures of ecosystem functioning (rather than just biodiversity features) [11]. Here, in addressing these limitations we document several key results.

First, there are general patterns of difference in NDVI, a surrogate of plant production, inside and outside protected areas. Where there are differences, NDVI tends to be higher inside forested protected areas, and vice versa for non-forested protected areas. This would suggest strong support for the conclusions of previous, typically local, studies showing that the designation and implementation of protected areas often acts to reduce levels of land use change and resource extraction [22], [24], [28]. That is, protected areas have a positive effect on the representation of plant production. This could occur for a variety of reasons, including the establishment of protected areas in regions in which plant production initially differed from that elsewhere, and changes in plant production as a consequence of management within protected areas. However, the greater NDVI within forested protected areas than outside seems most likely to have arisen because deforestation frequently occurs at markedly greater levels in the surroundings than within the core [22]–[24], [27]–[29]. A further comparison indicated that for forested protected areas, forest covers 81.0% and 63.6% of the core and surroundings respectively (t = 12.9, n = 550, p<0.001), and cropland covers 2% and 6.7% (t = −8.2, n = 550, p<0.001). Likewise, the lower NDVI within non-forested protected areas than outside is likely to have arisen because plant productivity in the surroundings is increased by irrigation and fertilization or overgrazing induced woody encroachment [59]–[62]. For example, cultivation increased the net primary production (NPP) in the US Great Plain by 10% [59]. For non-forested protected areas, cropland covers 4.5% and 6.2% of the core and surrounding areas respectively (t = −3.76, n = 465, p<0.001).

These interpretations of the results are also supported by the temporal patterns of change in NDVI (see below). The lack of significant differences in NDVI between protected areas and their surroundings for needleleaf forests (Fig. 2b) may be because human population density in these regions is typically particularly low (∼6.9 persons/km^2^), resulting in limited transformation of these surroundings. A similar result for wooded grasslands (Fig. 2b) may simply be because plant production is quite similar whether these environments are natural or strongly human-influenced (global mean NDVI of croplands  = 0.426 and mean NDVI of wooded grasslands  = 0.417), leading to little ability to discriminate between the two on this basis.

Second, differences between the NDVI of protected areas and that of their surroundings have been widely maintained over a 25 year period, during which the global human population has increased by ∼45% [63] and there have been substantial changes in patterns of global land cover [4], [64]. Indeed, across all of the protected areas examined and just for those forested or non-forested, and despite substantial annual variations, the differences have remained quite consistent (Fig. 5). This is one of the only explicit pieces of evidence to date for a widespread influence of protected areas on the persistence of ecological function.

The period 1982–2006 has seen a global increase in NDVI values of ∼2.7% (data not shown). This is thought principally to be a consequence of nitrogen deposition, CO_2_ enrichment fertilization, and climate change [65]–[67]. The increase has been sufficient that for non-forested protected areas (particularly shrublands; Fig. S1) the average NDVI found within their core in 2006 was greater than the levels observed in their surroundings in 1982, although that in the core remained substantially lower than that in the periphery throughout the period. This highlights the significant impact that processes operating on much greater spatial scales can have even on large protected areas [11] (all those included in the analysis were ≥500 km^2^). It also argues for consideration of some of these processes in establishing realistic management goals for protected areas, as it is doubtful that these broad changes in plant production could in the long-term effectively be opposed through management.

The third key result of these analyses is that although differences in NDVI between protected areas and their surroundings have largely been maintained over a 25 year period, for some land cover types the magnitude of the differences has significantly changed. That is, whilst protected areas have generally enabled the persistence of this ecological function relative to their surroundings, their effectiveness in so doing has been altering. For evergreen broadleaf forests and barren grounds there were net increases in the difference in NDVI between protected areas and their surroundings (Fig. S2). In the first case this results from an increase in NDVI within the protected areas and no directional change in the surroundings, likely because primary production was being enhanced in the former, following global trends, whilst in the latter continued deforestation offset any such gains [36]. In the case of barren grounds, NDVI within protected areas exhibited no significant temporal trend because of the very sparse vegetation coverage, whereas in the surrounding areas it increased significantly, in line with global trajectories [48]. These results highlight the fact that, unsurprisingly, whilst space-for-time swaps are commonly used in evaluating the effectiveness of protected areas, the differences between protected areas and their surroundings are (on many axes) often changing continuously and sometimes in complex ways.

In sum, these results suggest that over a quarter of a century protected areas have on average proven effective in the protection of plant production, a vital ecosystem function, in terms of the two key components of ecological effectiveness, their representation of this function and its persistence through time.

## Supporting Information

Figure S1 **Temporal trends in NDVI in the core (orange) and boundary (blue) of protected areas, and their surroundings (grey) for different land-cover types.** Solid fit line represents significant, and dashed for non-significant, at p<0.01.
(TIF)

Figure S2 **Temporal trends in δ- NDVI (difference of NDVI between core of protected areas and their surroundings) for different land-cover types.** Solid fit-line represents significant, and dashed for non-significant, at p<0.01.
(TIF)
