# Supplementary figures and images for: Effectiveness of Protected Areas in Maintaining Plant Production
Source: PLoS One. 2011 Apr 28;6(4):e19116. doi: 10.1371/journal.pone.0019116 (PMC3084248; doi:10.1371/journal.pone.0019116)

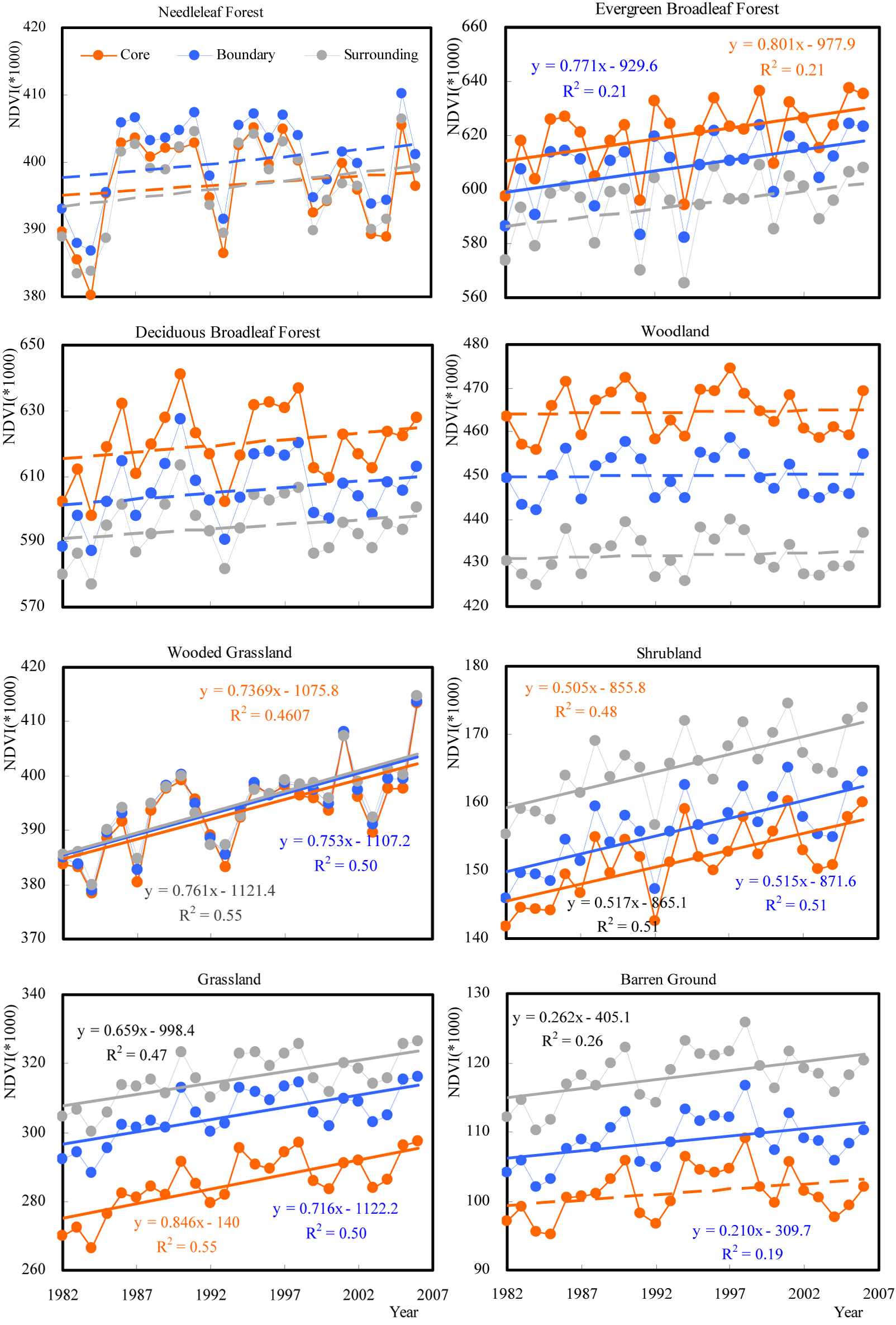

Supplement: Figure S1 — Temporal trends in NDVI in the core (orange) and boundary (blue) of protected areas, and their surroundings (grey) for different land-cover types. Solid fit line represents significant, and dashed for non-significant, at p<0.01. (TIF) [file pone.0019116.s001.tif]

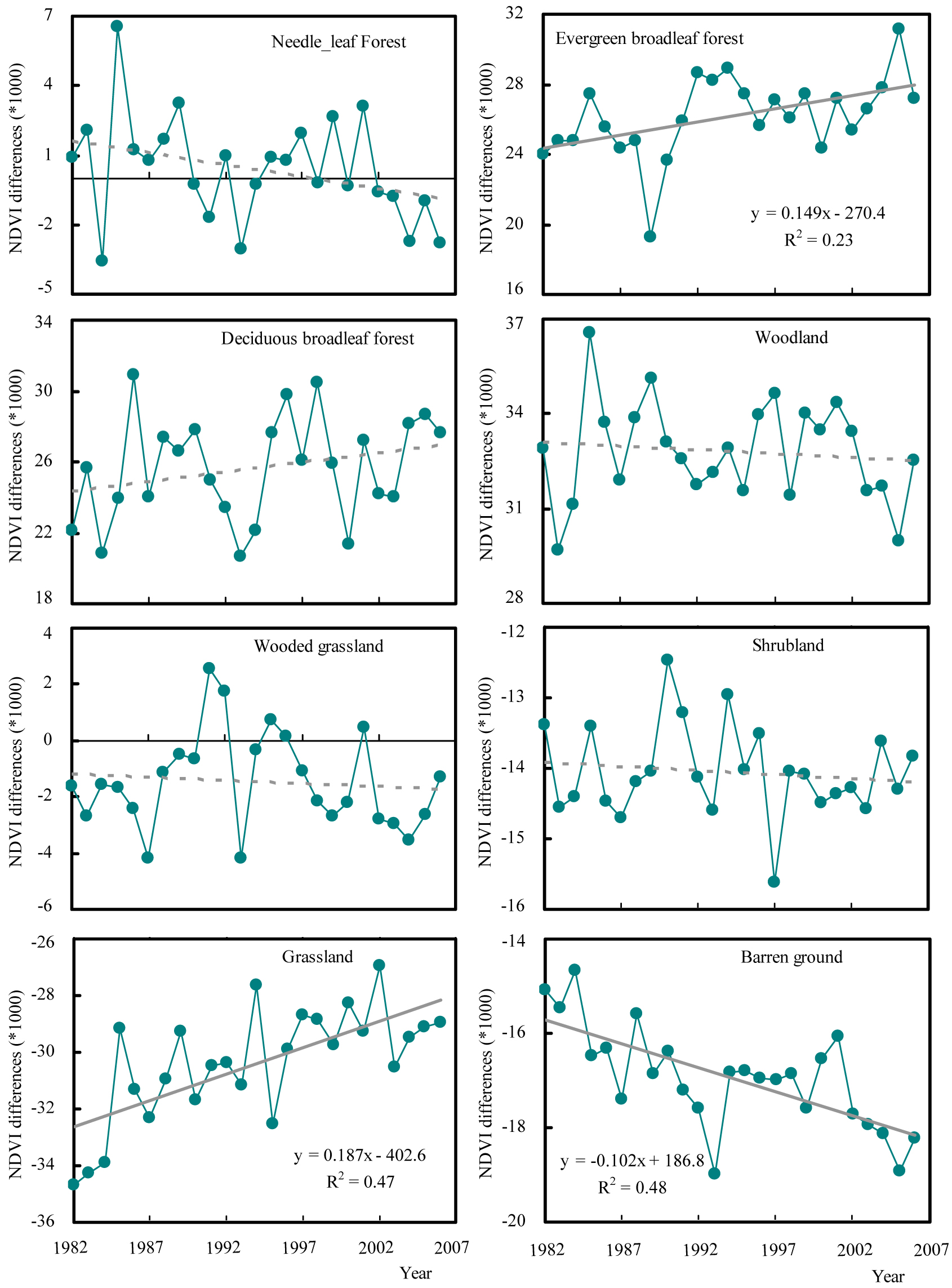

Supplement: Figure S2 — Temporal trends in δ- NDVI (difference of NDVI between core of protected areas and their surroundings) for different land-cover types. Solid fit-line represents significant, and dashed for non-significant, at p<0.01. (TIF) [file pone.0019116.s002.tif]
